# Faculty of Comparative Medicine and Veterinary Science

**Published:** 1893-01

**Authors:** 


*Faculty of Comparative Medicine and Veterinary Science.*—The regular fortnightly meeting of the Medical Association of the Faculty of Comparative Medicine and Veterinary Science took place in the College lecture-room, 648 Union Avenue, November 11th. Dr. Mills occupied the chair. There were also present Dr. D. McEachran, Dr. Johnson and Dr. Adami.

The routine business of the evening being transacted, Mr. Brainard was called upon to read his paper on "Texas Fever." The paper showed careful preparation, and was well delivered. Following is the synopsis of the main points touched upon:

This bovine malady causes great yearly losses to the cattle men of the South and West, also appearing frequently in the East. Fifty to seventy-five per cent. of Northern cattle shipped across the fever limit die of this plague, thus effectually closing the splendid Southern market to Northern cattle dealers, and, on the other hand, depriving the Southern cattle of the excellent pasturages of the North.

That these States must profit enormously if this disease could be eradicated is quite apparent; that it will be stamped out there is no doubt, if scientific and continuous investigations be maintained effectually.

By the help of various stock-raisers a series of experiments were arranged in 1887 at the Experimental Station in Missouri, under the direction of Dr. Paul Paquin (a former graduate of the Montreal Veterinary College, and an enthusiastic member of the Montreal Veterinary Medical Association), who had discovered a micro organism in the blood and tissues of diseased animals as early as 1885. The objects of these investigations were as follows:

1. To determine the location of the disease in the different States, also what it was that acted as a vehicle and preserved the vitality of the virus.

2. To determine the location of the virus in the animal body, and how it was transmitted.

3. To determine the accessory sources of transmission of virus, such as hay, ticks, food, etc.

4. The period of incubation of the disease in Northern cattle.

5. Experiments such as inoculation, etc., to render Northern cattle safe against Texas fever.

6. To determine whether calves born south of the fever limit were proof against infection by natural inoculation from mother before birth, or afterwards from the soil, or both.

One hundred and forty specimens were collected from different localities in the South of substances, in all of which germs were found except dry hay, subsoil, spring water, well water and one specimen of surface soil; manures were especially prolific.

Cultures were made and white mice inoculated, causing death only where a large quantity was used. The products of these cultures would not kill cattle with typical symptoms of Texas fever, although temperature sometimes ran as high as 107 degrees Fahrenheit.

Two important conclusions were arrived at by these experiments:

1. That the tissues of Southern cattle contained a species of germ, which was also in the young animal before birth, as well as in fæces, water and grounds where fever was located.

2. That the germ was absent in dry fodder, well water, spring water and subsoil.

The investigations were pushed during the summer of 1890, and some advances of great practical importance arrived at.

The summer of 1888 was spent in determining the presence of a specific microbe as the cause of the disease; this was important, as several investigators had attributed it to specific bacterium.

The investigations showed that the disease was not due to bacteria, but to a parasite which inhabited the red blood cells, and whose presence finally caused dissolution of same.

This discovery explained the symptoms of the disease, as well as the cause of the constantly diminishing number of red blood corpuscles.

The Southern cattle are infested with ticks, which, when full grown, drop off, lay their eggs on the grass, and perish. The young hatch out in from fifteen to thirty days, get back upon the cattle, mature, and then drop off again, going through the same routine as the preceding crop. This is kept up till cold weather sets in.

It had been suggested that ticks were the cause of the fever in Northern cattle from the fact that the disease never appeared until the ticks had attached themselves to the bodies of the animals.

This theory was put to the test by Dr. Kilborn, who picked the ticks from a herd of Northern cattle as soon as they were large enough to be detached from the skin ; then keeping the pasture free from infection ; at the same time another herd of Northern cattle infested with ticks were kept in a separate enclosure from the first herd. The latter all died from Texas fever, while the former showed no signs of the disease. The next experiment was as follows :

Two different fields were selected, the first to contain North Carolina cattle with ticks; second, Texas cattle with ticks; third, North Carolina cattle without ticks; fourth, ticks only; fifth, soil from infested fields. The following result was obtained : The first animal to die was in the field containing North Carolina cattle with ticks. No death occurred from the soil field.

This experiment proved that the ticks must be in actual contact with the bodies of the cattle, and that Southern cattle were exempt from the disease. The way the ticks produce the disease is apparently as follows : The ticks drop off the cattle when they come to maturity, carrying with them the germ of Texas fever with which they infest the grass. This virus takes from 30 to 60 days to ripen, as Northern cattle when placed in the same enclosure with Southern, do not show any signs of the disease until from one to two months after exposure. Thus the period of incubation of the disease is explained by the life history of the tick. Taking all these proofs together, we come to the conclusion that where there are no ticks there is also an absence of Texas fever. Symptoms: Elevation of temperature may run as high as 107 degrees Fahr., pulse soft and quiet. Drooping ears, sluggish gait, deficiency of secretions; lameness in one or more limbs. The appetite may return and rumination may take place days after the disease has gone on and disturbed the functions of the other important organs. The urine becomes scanty and high colored, probably due to the presence of Hæmoglobus urea caused by the breaking down of the red blood corpuscles, setting free the Hæmoglobin which is secreted by the kidney; bloody fæces and diarrhœa in many cases; animal evinces great pains; comatose condition occurs in last stages; if brain is effected, delirium.

Before death takes place temperature often falls to 93 degrees Fahr.; death takes place in from one to seven days after first symptoms appear.

Post mortem appearances: *Blood.* Bright red, thin, and coagulates quickly; great lack of red cells, about one-sixth normal number.

*Flesh.* Muscular tissue has a bright clear appearance: the fat has a brownish tint.

*The Spleen.* Five or six times normal size and when cut across presents a disintegrated appearance.

*Liver.* Diagnostic of this disease; yellowish brown in color, enormously enlarged, parenchyma deeply stained with bile. The interlobular biliary system is engorged with blood, also have parenchymatous degeneration of a fatty nature.

*Gall Bladder.* Always distended with gall; walls being hypertrophied; color of gall varies, from yellow to green and sometimes black.

*Alimentary tract.* Lesions found in abomasum and rectum; mucous membrane dark red and congested; when diarrhœa is present the rectum is the seat of severe hemorrhages.

*Kidneys.* More or less congested.

*Bladder.* Usually distended with highly colored urine.

*Lungs.* May be emphysemetous, with hypostatic congestion, pleurisy may or may not be present.

*Treatment.* Any treatment bestowed on cattle suffering from Texas fever is useless.

*Prevention.* Inoculation the only known prevention. Dr. Paquin has demonstrated that inoculated animals can be shipped across the fever limit with impunity.

At the conclusion of this excellent paper considerable discussion ensued with regard to "ticks," "fever limit," etc.

The President asked Mr. Brainard "how he considered the cold effected the reduction of the fever?" "By killing the ticks," replied Mr. Brainard. A member: "What is the Northern limit of Texas fever?" Mr. Brainard: "It is not constant but is continually moving Northward, at present about 37 degrees."

An interesting case of Sarcoptes Equi was reported by Mr. Tracy; after this had been discussed the meeting adjourned.

